# Addressing conflicts of interest regarding the vaccine in infectious disease outbreaks based on good governance for health approach: a policy brief

**DOI:** 10.1186/s12913-023-10020-w

**Published:** 2023-09-25

**Authors:** Nazanin Soleimani, Hamed Ghoshouni, Hakimeh Mostafavi, Mohammad Hossein Modiri, Mohammad Hasan Movahedian Attar, Seyed Masood Mousavi

**Affiliations:** 1 Health Management and Economics, School of Public Health, Health Policy and Management Research Center, Shahid Sadoughi University of Medical Sciences, Yazd, Iran; 2 Health policy Research center, Institute of Health, Shiraz University of Medical Sciences, Shiraz, Iran; 3 Health Equity Research Center, Tehran University of Medical Sciences, Tehran, Iran; 4 School of Medicine, Shahid Sadoughi University of Medical Sciences, Yazd, Iran; 5 Department of Medicine, School of Medical Sciences, Yazd Branch, Islamic Azad University, Yazd, Iran

**Keywords:** Vaccine, Conflicts of interest, Infectious disease outbreaks, Good governance for health

## Abstract

**Background:**

Infectious disease outbreaks pose a significant threat to public health, and achieving herd immunity highlights the importance of addressing conflicts of interest (COI) in vaccine development and policy-making. This policy brief aims to present policy options that address COI regarding vaccines in infectious disease outbreaks, based on good governance for health approach.

**Methods:**

Our study used a scoping review methodology. We conducted a systematic search, which led to identifying 43 eligible articles. A qualitative approach (i.e., content analysis) was employed for data analysis, using “ATLAS.ti 9” software. The primary results underwent a process of cleaning, categorisation, and subsequent discussion in three sessions with the research team.

**Results:**

Relationships between theindustry and “government/policymakers” as well as "academic institutions/researchers" are prominent origins of COI regarding the vaccine in infectious disease outbreaks. To address this issue, we present nine policy options that target both the root cause of the problem and the adoption of good governance for health approach.

**Conclusions:**

The key principles of good governance for health, including, “Transparency”, “The Rule of Law”, “Effectiveness”, “Efficiency”, “Participation”, “Consensus Orientation”, “Equality”, “Responsibility”, “Responsiveness” and “Accountability” must be taken into account when formulating policy options to address COI regarding the vaccine in infectious disease outbreaks. The effectiveness of the policy options outlined in this policy brief should be assessed in practical contexts, as this evaluation may uncover the need for revisions.

**Supplementary Information:**

The online version contains supplementary material available at 10.1186/s12913-023-10020-w.

## Background

Outbreaks significantly threaten public health and health policy-making [1]. The COVID-19 pandemic, one of the most critical crises in the last 50 years, has claimed millions of lives; As of July 5, 2023, the World Health Organization (WHO) COVID-19 dashboard reports a total of 6,948,764 deaths [2]. Vaccination and achieving herd immunity are crucial in combating infectious disease outbreaks [3, 4]. Within the medical field, discussions on vaccination have consistently been contentious, with various groups accusing one another of conflicts of interest (COI) [5, 6]. It is worth acknowledging that the necessity of achieving herd immunity, coupled with the substantial financial contracts involved in vaccine production, can create an environment susceptible to the influence of COI on vaccine development, guidelines, and policy-making [7]. Building Public trust serves as a fundamental pillar for successful vaccination campaigns. However, the low utilisation of vaccines such as measles, tetanus, and COVID-19 in various countries [8–13]. indicates a decline in public trust towards scientific research and the guidelines provided by health organisations, which raises serious concerns [14, 15]. A significant factor contributing to this decreased public trust is the presence of COI within the vaccines field, leading to corruption in vaccine production and development and related policies [7, 16–23]. As a result, one of the primary arguments by anti-vaccine groups revolves around the perceived COI of experts, researchers, and policymakers due to their financial connections with the industry [7]. Other factors contribute to the concern surrounding the potential detrimental effects of industry relationships on public health decisions. For instance, in the swine flu pandemic in 2009, there were debates about the adequacy of evidence supporting the declaration of a pandemic by the WHO and their prediction of infection in nearly two billion individuals [24]. Notably, vaccine production companies, as the primary stakeholders, reaped substantial benefits from the situation [25].

COI arise when public officials face a conflict between their public duties and private interests, where their private-capacity interests can potentially exert undue influence on fulfilling their official responsibilities [26]. Corruption, as defined by the World Bank, is the misuse of public office for personal and private interests [27]. Therefore, while COI can potentially lead to corruption, it is essential to acknowledge that corruption consistently stems from COI [28].

Research has demonstrated that existing policies about identifying and mitigating COI in developing and evaluating vaccines [28–30] and policies addressing the COI of decision-making bodies are insufficient and require bolstering [24, 31–38].

In Iran, notable policy measures specifically targeting COI regarding the vaccine during the COVID-19 pandemic were not formulated by the National Headquarters for COVID-19 and the Ministry of Health and Medical Education. Specific individuals who held positions within these organisations were also involved in vaccine development teams, including the “Barakat vaccine”, which sparked considerable discussions surrounding COI [39].

It is crucial to prioritise public health over industry interests, address COI in vaccine research and evaluation, and develop vaccine instructions and policies. These efforts prevent corruption, foster public trust, and enhance public health outcomes. Therefore, based on a scoping review, this policy brief aims to provide policy options for effectively addressing COI regarding the vaccine in infectious disease outbreaks. It takes the good governance for health approach, incorporating principles such as “Consensus Building’, “Participation”, “Transparency”, “Equality”, “The Rule of Law”, “Effectiveness and Efficacy”, “Responsibility”, and “Accountability” [40].

## Methods

This study utilises the scoping review method, which is suitable for addressing “what” and “why” questions. As described in the definitions provided for scoping review, this approach is specifically designed to swiftly examine critical and fundamental concepts within a particular research field while identifying key sources and evidence. In this study, we employed the protocol^1^ developed by O'Malley and Arksey to carry out the scoping review [41].

The research team searched international (Web of Science, Scopus, PubMed, and EMBASE) and national databases (MagIran and SID) to identify relevant studies from the inception of these databases until August 2022. We also mined Google Scholar to increase the chance of finding potentially relevant studies on the topic under scrutiny. Additionally, we searched Grey literature and thesis. The search was conducted in English and Persian languages. The reference list of the selected articles was hand-searched to increase the chance of not missing potential articles. We provide the search strategy for each database in (Appendix 1). Epidemic, pandemic, vaccine, conflicts of interest, outbreak, fraud, and corruption keywords were used to search the international databases. Additionally, the Persian counterparts of these keywords were employed to search national databases. All stages of the search process were conducted independently by two researchers.

Inclusion criteria for this study encompassed research articles written in English and/or Persian that provided examples or solutions addressing COI regarding the vaccine in the context of infectious disease outbreaks. Conversely, studies that lacked relevance to the research topic or offered incomplete information were excluded based on the exclusion criteria.

The articles underwent screening based on the PRISMA framework [42]. Initially, duplicate articles were eliminated. Subsequently, two independent researchers screened the titles and abstracts of all articles based on the predefined inclusion and exclusion criteria. In line with the anticipated objectives of this article, any studies considered irrelevant were excluded from further analysis. In the following stage, articles that were considered relevant based on their title and abstract, as well as those for which the determination of relevance solely based on title and abstract was not feasible, were selected for full-text retrieval. Subsequently, two individuals reviewed these articles, and those that closely corresponded with the objectives of our study were included for further analysis. In instances of disagreement, consensus was achieved through discussions, and if needed, a third reviewer was consulted to facilitate resolution. Initially, the search strategy yielded a total of 1,635 articles, of which 80 were duplicates.

Consequently, the title and abstract of 1,555 articles and the full text of 223 articles were assessed, resulting in the inclusion of 43 eligible articles (Appendix 2). Then, we subjected the included articles to quality appraisal, using a specific assessment checklist for each category: “The JBI Critical Appraisal Checklist for Text and Opinion Papers” for evaluating theoretical-analytical, commentary, editorial, and news articles [43] “qualitative CASP checklist” for evaluating qualitative studies [44]; and, the “CASP checklist for review” for evaluating review articles [45]. Due to the limited literature on the topic, excellent and average-quality articles were included. Following the extraction of primary data, eligible articles were assessed to address the following inquiries:

1. What was the primary objective and methodology employed in the study?
2. What bottlenecks of COI regarding the vaccine in infectious disease outbreaks were discussed in this study?
3. What solutions were implemented to address COI regarding the vaccine in infectious disease outbreaks?
4. What were the advantages, disadvantages, and practical considerations associated with the mentioned strategies for addressing COI regarding the vaccine in infectious disease outbreaks?

Data analysis was conducted using a qualitative approach (i.e., content analysis) utilising ATLAS.ti9 software. Themes and sub-themes were extracted from the data and underwent a review, refinement, and classification process. Through this process, bottlenecks and inferred policy options were identified. In three sessions with the research team, we followed these steps: Similar bottlenecks of COI and inferred policy options were grouped. Next, based on the identified origins of COI regarding the vaccine in infectious disease outbreaks, nine policy options were categorised into three distinct categories.

## Results

Based on the scoping review conducted by the research team, the prominent cases of COI regarding the vaccines in infectious disease outbreaks were found to be originated from “relationships with the industry”. This category can be further divided into two subcategories:

1. Relationships between the industry and government/policymakers.
2. Relationships between the industry and academic institutions/researchers.

After reviewing articles and available evidence, ten bottlenecks of COI regarding the vaccine in infectious disease outbreaks were identified. These bottlenecks were categorised into two groups based on their origin, as outlined in (Table 1). Inferred Policy options derived from the literature review to address these bottlenecks are presented in (Table 2).

**Table 1:** Origin and bottlenecks for COI regarding the vaccine in infectious disease outbreaks extracted from the literature review

Bottlenecks	
**Relationships between the industry and government/ policymakers**	**Relationships between the industry and academic institutions/ researchers**
1) COI in budget allocation and negotiation for the production and import of vaccines and drugs in infectious disease outbreaks [46–48].	8) COI in development and updating guidelines for vaccines and vaccination in infectious disease outbreaks [37].
2) COI in decision-making and planning for vaccination in infectious disease outbreaks.	9) COI in the development, implementation, publication, and rejection of projects on the discovery, efficacy evaluation, and effectiveness of vaccines [6, 30, 49–51].
3) COI in the licensing process of vaccines in infectious disease outbreaks [31, 52–54].	10) COI of advisory committees’ members providing evidence to policymakers and officials [31, 34, 36].
4) COI in collaboration between private and public sectors for post-administration monitoring of vaccines [52–55].	
5) COI of members of the promotion and marketing committee of vaccines [56].	
6) COI in the field of the supply chain, expansion, and quality control of manufactured vaccines in infectious disease outbreaks [46, 57–59].	
7) COI in the access of particular individuals and various groups to vaccines [46].

**Table 2:** Inferred Policy options derived from the literature review

Inferred Policy Options^a^	
1	Enhancing the monitoring, evaluation, and accountability of policymakers’ decisions regarding vaccines in infectious disease outbreaks while establishing appropriate penalties for any violations of the law [11, 46].
2	Conducting a comprehensive review of existing anti-corruption laws and policies and implementing measures to strengthen them further [48].
3	Creating a collaborative decision-making body comprised of diverse stakeholders, including universities, the pharmaceutical industry, the health sector, and legislative bodies, to effectively address the impacts of COI [46, 55].
4	Establishing an independent inter-sectoral regulatory body composed of impartial members to oversee the authorisation of vaccine administration [53].
5	Ensuring transparency and disclosure of COI cases for all members of policy-making and decision-making committees [31, 60].
6	Disclosing COI of technical committees’ members regarding the meeting’s topic at the beginning of the meetings [34].
7	Publishing the list of authors and funding sources for developing vaccine guidelines and publicly disclosing any associated COI cases [61].
8	Implementing prohibition on the engagement of professionals with COI in the development of vaccine-related guidelines [61].
9	Requiring researchers to annually disclose their vested interests, which have the potential to create COI, using a standardised form [30, 51].
10	Outlining the process of disclosing, updating, and managing potential COI within the project contract and ensuring researchers sign the contract before commencing the research [30].
11	Standardising and developing protocols for the disclosure of COI in vaccine guidelines [37].
12	Establishing an inclusive and transparent framework for making decisions related to vaccines, ensuring openness and accountability throughout the process [52].
13	Enforcing a prohibition on the involvement of individuals with COI in vaccine-related policy-making processes [25, 34, 55].
14	Implementing a prohibition on the membership of individuals involved in the development of domestic vaccines in decision-making and policy-making committees responsible for the procurement of foreign vaccines [62].
15	Conducting training programs for policymakers and decision-makers to raise awareness and promote a shared understanding of COI [63].
16	Developing protocols to address COI in vaccine technical advisory committees [33].
17	Implementing transparent and accountable public emergency procurement practices by utilising open contracts and electronic procurement methods [46].
18	Strengthening the media's role by providing support for monitoring and clarifying COI cases and advocating for policy-making aimed at effectively addressing COI [64, 65].
19	Raising awareness and strengthening advocacy by informing society about COI cases through websites and social media platforms [66].
20	Establishing a platform to facilitate the engagement of civil society in decision-making processes concerning vaccines, enabling their participation through various communication channels such as mobile software and hotlines for reporting and feedback [46].
21	Providing support to individuals who report COI cases and safeguarding accountable organisations and individuals from political pressures and influences [11, 46].
22	Establishing a dedicated committee to oversee emergency funding allocation and the deployment of vaccines [46].
23	Clarification and documentation of high-level decisions regarding contracts by bypassing instructions and regulations such as contracts for purchasing vaccines in emergency time [36].
24	Designing innovative local mechanisms and robust regulatory systems to monitor the supply chain and ensure the quality of vaccines [59].
25	Clarification and documentation of governmental decisions regarding contracts while bypassing formalities and regulations, such as contracts for the procurement of vaccines in emergencies [36].
26	Developing and utilising strategic frameworks, pre-designed national and international guidelines, and specific criteria throughout various stages of financing and allocating vaccines in infectious disease outbreaks [46].
27	Implementing robust enforcement mechanisms and stringent penalties within the legal framework to prevent corruption in vaccine manufacturing, ensuring severe consequences for any violations [57].
28	Identifying high-risk relationships and interests and establishing standardised protocols for the selection of committee members, excluding or requiring abstention from those with COI [35, 60].
29	Following a standard protocol for consensus building in the development of vaccine-related guidelines [37].
30	Enhancing the clarity of the COI section in guideline assessment tools and having evaluators investigate the COI cases of guideline developers [37].
31	Enhancing the role of research ethics committees in promoting and overseeing research conducted under ethical principles [49, 67].
32	Establishing a platform for open scientific discussions on the safety and effectiveness of various vaccines while actively monitoring journals to ensure the publication of articles encompassing both positive and negative results [6].
33	Developing proactive management strategies to address COI cases in the vaccine research team effectively [51].
34	Implementing an alert banner on preprint servers for articles that are not peer-reviewed and lack clear disclosure COI [49].
35	Creating a central entity responsible for receiving reports on potential cases of fraud in vaccine research and disseminating information about retracted studies to affected organisations and individuals [50].
36	Implementing robust regulatory policies by journals to prevent the manipulation of vaccine research data [29].
37	Utilising blockchain technology as a safeguard to protect the integrity and security of vaccine research data [29].
38	Leveraging blockchain technology to establish a framework that promotes data transparency, immutability, and efficient vaccine registration processes, thereby mitigating the risks of counterfeiting and identity theft [58].
39	Leveraging blockchain technology to establish a vaccine supply chain management framework that incorporates anti-manipulation and fraud detection mechanisms, ensuring transparency and integrity throughout the process [58].
40	Developing and implementing robust tracking systems for secure storage and distribution of vaccines to mitigate the risks of expiration and unauthorised diversion to the black market [46].
41	Identifying and providing support to vulnerable groups and communities that are most impacted by corruption, ensuring they receive necessary assistance and protection [46].

^a^The policy options are deduced or derived from the information presented in the papers, even if they are not explicitly mentioned as policy options

Based on the research team sessions, the policy options for addressing COI regarding the vaccine in infectious disease outbreaks, as categorised by origin and approach to strengthening good governance for health, are as follows:

- Common policy options:
  1. Promoting the disclosure and transparency of COI for involved individuals and organisations along with the standardisation of disclosure procedures;
  2. Restricting or excluding those with COI from participating in technical and policy committees;
  3. Providing training to decision-makers and researchers, facilitating dialogue, and promoting a shared discourse on COI;
  4. Revising and enhancing current anti-corruption laws and policies and establishing legal sanctions for violations;
- Policy options to address COI arising from the relationships between the industry and government /policymakers:
  5. Strengthening monitoring, evaluation, and accountability mechanisms;
  6. Empowering the community and news media for advocacy;
  7. Establishing an intersectoral and multisectoral decision-making body comprising diverse stakeholders;
- Policy options to address COI arising from the relationships between the industry and academic institutions/ researchers:
  8. Developing Policies on data protection, publication, and retraction of vaccine research;
  9. Establishing standardised procedures and protocols for conducting studies and developing vaccine guidelines;

## Description of policy options

### Common policy options

#### 1. Promoting the disclosure and transparency of COI for involved individuals and organisations along with the standardisation of disclosure procedures

The true importance of public disclosure lies in its capacity to foster accountability within relevant committees, thereby empowering the public to assess the alignment of a committee's actions regarding COI with its stated intentions (60). Any actual or potential COI must be disclosed, made accessible to the public, and promptly updated if any changes arise. Promoting public transparency enables the continuation of productive and beneficial endeavours and serves as a deterrent against improper relationships. It is a cautionary message from the public to those with COI [60].

While transparency is essential in preserving public trust, it alone is insufficient. In situations where the underlying context is poorly understood, disclosures can become colourful descriptions of numerous and varied business relationships [68]. In addition, disclosures are often missed, incomplete, inconsistent, and unavailable [34]. Overemphasising disclosure may also lead researchers and policymakers to develop a false sense of moral immunity, absolving them of their responsibility to address COI. The disclosure of COI fails to provide transparent information about the intensity and impact of those conflicts on the public. Consequently, it fails to disclose the underlying interests upon which the decisions and policies are based [60]. This failure becomes particularly concerning when minutes of meetings and the perspectives of each person involved are unclear, making it difficult for people to assess the extent to which COI may affect an individual's views. Therefore, it is crucial to have apparent, measurable, clarified, and standardised primary interests accompanied by precise definitions for COI [32].

Additionally, an independent committee should continuously evaluate and validate the standards related to COI [60]. In situations like infectious disease outbreaks, where vaccine purchase and production contracts are hastily concluded due to emergency conditions, bypassing predetermined laws and formalities, or before thorough research on vaccine effectiveness and safety is completed, government decisions must be appropriately documented and clarified. This documentation and clarification ensure that the decision-makers’ interests do not inappropriately influence the outcome [36]; Moreover, in cases where concerns arise about the impact of COI on decisions (perceived interests), official organisations should promptly and transparently report to the public without resorting to defensive statements, in order to maintain public trust [69, 70].

#### 2. Restricting or excluding those with COI from participating in technical and policy committees

According to guidelines from organisations like EMA (European Medicines Agency), OECD (Organization for Economic Co-operation and Development), and WHO, effective management of COI involves identifying and mitigating risks rather than systematically excluding stakeholders with COI. Meanwhile, in some situations, COI may limit the involvement of experts, either individually or organizationally, in particular decisions or actions. However, if these professionals can contribute in other beneficial ways unaffected by their COI, there may be no need to exclude them from the entire project [55].

Expert committees should establish clear criteria for defining high-risk interests or relationships, select members and voting rights based on consistent and transparent standards Preference should be given to those without COI or individuals willing to set aside their interests [60]. In cases where there is no alternative but to involve individuals with COI (e.g., due to their specialised expertise), engaging their services in an independent advisory committee is possible by developing innovative strategies [24, 60, 69].

#### 3. Providing training to decision-makers and researchers, facilitating dialogue, and promoting a shared discourse on COI

Due to ongoing debates regarding the approach to address COI and the normalisation of the relationship between industry, academia, and governance, it is not unexpected that policymakers and academics may still require further clarification on which types of relationships give rise to COI. Insufficient understanding of the ethical basis of COI appears to be prevalent [63]. Therefore, fostering dialogue and familiarising policymakers and academics with the existing COI literature is essential, presenting them with concrete cases exemplifying the negative consequences that can arise.

#### 4. Revising and enhancing current anti-corruption laws and policies and establishing legal sanctions for violations

Revisions of laws should ensure the active participation of civil society, the rule of law, effective management, transparency, and accountable governance [46]. Additionally, it is crucial to ensure the enforcement of laws to prevent corruption among vaccine manufacturers by imposing legal sanctions. Possible measures include facility inspections, product seizure, trade limitations (e.g., legal suspension of business activity), professional development training (e.g., regulatory and business ethics training courses), and import bans [57].

### Policy options to address COI arising from the relationships between the industry and government/policymakers

#### 5. Strengthening monitoring, evaluation, and accountability mechanisms

Few countries [71], like the Republic of Korea, Singapore, China, and Egypt have National Regulatory Authorities (NRAs) classified by the WHO as advanced or functional and integrated regulatory systems (Maturity level 3 or 4 based on the WHO Global Benchmarking Tool (GBT)) [72] that protect the majority of the population, particularly against substandard and counterfeit medical products.

To strengthen supervision, developing and implementing national and local strategies and programs to combat corruption, including in emergencies, is necessary by converting knowledge into concise and targeted solutions. International guidelines can be utilised for this purpose [46].

Establishing a committee to assess quality and conduct audits from the early stages of infectious disease outbreaks is crucial. This committee enables the evaluation and addressing COI. The impact of COI on research and governance functions should be assessed at both individual and organisational levels through a transparent COI management program overseen by the quality control and audit committee [55]. Public institutions can employ corruption risk assessment to identify vulnerabilities in their operations and develop practical and cost-effective strategies to mitigate them. Additionally, it is recommended to establish a dedicated committee to monitor emergency funds and vaccine deployment. This committee should encompass monitoring capabilities for emergency funds, vaccine procurement and distribution, and related processes in real-time to promptly identify any risks [46].

Local innovative mechanisms like the African Vaccine Regulatory Forum could contribute to addressing emergencies such as COVID-19 [59]. Also, new technologies such as blockchain can enhance data transparency and vaccine supply chain management. Establishing tracking systems for secure vaccine storage and distribution is advisable to strengthen monitoring. This system includes safeguarding vaccines in undisclosed locations and closely monitoring their transportation, thereby minimising corruption and diversion risks [46].

#### 6. Empowering the community and news media for advocacy

To empower community advocacy, establishing a transparent central system accessible to the public is essential [66]. However, sharing a list of COI cases with the public is ineffectual and undermines trust in policymakers. Instead, it is crucial to address identified COI and provide clear explanations for the presence of individuals with COIs in decision-making committees. Furthermore, public awareness should be increased regarding the distinction between COI and corruption, emphasising communication benefits between researchers, industry, and the government. Additionally, creating a platform for civil society participation, utilising mobile software and hotlines can enable reporting and decision-making on vaccines. Developing a secure, effective, and gender-sensitive reporting system, such as anonymous proxies, is recommended [46].

During crises, the media is vital in revealing realities through investigative journalism and critical reporting on government policies. This role helps prevent COI cases that may divert crisis management from public health to political-economic fields, potentially resulting in corruption [64]. Establishing a public registration and publishing platform to disclose the COI of researchers and policymakers reminds journalists that COI can also influence news sources. This initiative can encourage journalists to expose COI cases [65]. Strengthening independent media and non-governmental organisations is necessary to prevent media COI and ensure impartial coverage of COI-related news.

#### 7. Establishing an intersectoral and multisectoral decision-making body comprising diverse stakeholders

Emphasising the establishment of a collaborative decision-making body inclusive of various stakeholders, such as academics, the pharmaceutical industry, the health sector, and legislative institutions, is crucial. This approach helps prevent undue influence from any single stakeholder and mitigates the negative impact of COI [6]. In addition to the decision-making institution, all decisions should be based on evidence and made in meetings attended by experts from the Ministry of Health, people’s representatives, and other relevant stakeholders. Transparency and accessibility of the decision-making process should be prioritised, with no room for ambiguity. Furthermore, a mechanism should be in place to update decisions based on emerging evidence, ensuring clarity and transparency [53].

### Policy options to address COI arising from the relationships between the industry and academic institutions/researchers

#### 8. Developing policies on data protection, publication, and retraction of vaccine research

Journals that publish basic vaccine research and clinical trials on vaccine efficacy and safety should emphasise the mandatory provision of raw data and enhance the scrutiny of research data, research protocols, and statistical conclusions. Also, they should carefully review the authors’ COI. Furthermore, the data management systems utilised in the studies relied on conventional data sources, lacking the ability to guarantee data immutability throughout the data generation and analysis stages. To address this issue, the adoption of blockchain technology can be considered. This innovative technology prevents data manipulation and enables transparent tracking of data modifications. Consequently, publications, journals, and stakeholders should require researchers to utilise blockchain-based systems and establish the necessary infrastructure [29].

To ensure confidentiality and prevent scientific misconduct and fraud, research should be grounded in good practice and ethical principles [67]. However, it is essential to note that while emphasising ethical principles preserves professional values, these principles do not possess legal and executive guarantees, and ensuring adherence by all researchers is challenging.

Another measure to address COI in the publication of vaccine-related research is to adopt practices similar to the bioRxiv preprint server and other relevant platforms. These servers display a yellow warning banner atop all new COVID-19 studies, serving as a reminder that these articles are preliminary reports and have not undergone peer review [49]. Another critical aspect to discuss in this context is the retraction of articles. The current mechanisms primarily focus on reporting potential fraudulent cases and retracting incomplete articles. However, there is a notable lack of transparency in writing the reports, identifying recipients, and ensuring proper follow-up to notify affected groups about the retracted article. To address this gap, it is crucial to establish a robust system that includes a centralised body capable of reporting suspected cases of fraud in vaccine research and effectively informing organisations and individuals affected by retracted articles. This system should be built upon a well-connected network of contacts and nodes, with prompt identification of network members. Any articles retracted due to fraud should be publicly announced to ensure transparency and accountability [50].

#### 9. Establishing standardised procedures and protocols for conducting research and developing vaccine guidelines

The development of guidelines should rely on valid handbooks to prevent potential harm to patients and the healthcare system caused by misinformation and misunderstandings. The GRADE (Grading of Recommendations Assessment, Development, and Evaluation) approach is a robust tool that can systematically be employed as a standard for developing vaccine guidelines. This approach involves three main stages: assessing the quality of evidence, comparing favourable and unfavourable effects, and determining the strength of the recommendation [35]. Noteworthy, following the instructions for developing vaccine-related guidelines should be mandatory and legally required for publication and approval. Simply recommending adherence to these guidelines would not carry sufficient weight. Guideline evaluators should utilise tools like AGREE and RIGHT to assess the quality of reporting and methodology in vaccine guidelines. Revising COI sections within the guidelines is crucial, enhancing their clarity.

Moreover, journal editors and guideline evaluators must establish a platform that facilitates open and impartial discussions, enabling the publication of research that examines both the support and challenges surrounding the safety and efficacy of vaccines. This platform should function independently and cross-departmentally, ensuring minimal COI and promoting an environment of free and unbiased scientific discourse [6]. Establishing an evaluation committee and involving multiple evaluators can be an effective strategy to achieve this objective. Vaccine research contracts and guideline development should also incorporate a COI declaration and establish a process to update and address potential COI. All research group members must disclose any interests that could lead to COI using a standardised form. Furthermore, the study’s final report should transparently publish information about financial sources, affiliations, and any possible COI, along with the results [37].

## Discussion

This policy brief, based on a scoping review study, highlights the bottlenecks and COI cases regarding vaccines in infectious disease outbreaks that demand the attention of policymakers and health sector managers. As previously mentioned, ten bottlenecks rooted in the “relationships with the industry” were identified and categorised as “relationships between the industry and government/ policymakers” and “relationships between the industry and academic institutions/ researchers”.

COI stemming from the “relationships between industry and academic institutions/researchers” can result in vested interest, leading to inadequate attention to the public interest and biased technical recommendations. These COIs can further influence research and guidelines, leading to ineffective outbreak management and resource wastage, ultimately risking lives. Similarly, COI arising from the “relationships between the industry and government/ policymakers” regarding budget allocations for vaccine import or domestic production, licensing, and post-market services can lead to inappropriate budget allocation, resource wastage, incorrect licensing decisions, delayed vaccination, and insufficient monitoring of vaccine safety, efficacy, and supply chain quality.

The consequences of COI, regardless of its origin, include a trust crisis and weakened health governance foundations. This weakness, in turn, contributes to vaccine hesitancy, hindering immunisation efforts and impeding the control of infectious diseases. The COVID-19 pandemic has demonstrated the compromise of public trust in scientists, governments, the healthcare system, and other related organisations, posing a significant challenge to accepting public health measures and vaccination [73]. Trust in public health interventions, including vaccines, relies on various factors and is closely linked to the institutions’ credibility in their development, approval, and administration [74–76].

In part, the challenge of addressing vaccine hesitancy can be attributed to the limited measures taken to align the interests of professionals, vaccine manufacturers, governments, and the public interest [77]. Vaccine manufacturers have not taken explicit and practical actions to build trust within societies. As a result, it is crucial to collectively work towards reevaluating the norms governing vaccine discovery, research evaluation, and vaccination decision-making; upholding the integrity and accuracy of vaccine research and policy-making demands significant effort and a transformative shift in communicating with the industry.

In our scoping review, we have identified several documents encompassing international experiences that are either fully or partially relevant to addressing COI regarding the vaccine in infectious disease outbreaks. These experiences have been remarkable and worthy of highlighting. Two of these experiences are related to The Innovative Medicines Initiative’s Accelerated Development of VAccine benefit-risk Collaboration in Europe (ADVANCE) consortium. The first experience is the “Guidance for the governance of public–private collaborations in vaccine post-marketing settings in Europe”. This guidance was developed after the 2009 influenza pandemic based on the need for appropriate infrastructures to strengthen public–private collaborations (PPCs), improve stakeholder interactions, and enhance the collection and analysis of safety and effectiveness data [55]. The ADVANCE consortium also released a “Code of Conduct for collaborative vaccine studies”. The development of this code of conduct was guided by three core values: best science, strengthening public health, and transparency. It involved a thorough review of existing guidance and relevant published articles. The ADVANCE Code of Conduct includes ten topics, including COI [30]. Another significant document is a supplementary document about the vaccine, which contains detailed descriptions of the experiences and processes of 15 well-established National Immunization Technical Advisory Committees from all world regions. These committees are crucial in providing national governments with information for evidence-based decisions regarding vaccine and immunisation policy [33]. These documents highlight the importance of addressing COI regarding the vaccine, establishing effective governance, promoting transparency, and making evidence-based decisions in vaccine-related collaborations and policy development during infectious disease outbreaks.

In Iran, there is a growing focus on COI within the health system, as evidenced by official documents and papers addressing COI (for example Islamic Parliament Research Center (IPRC) report on COI in the health sector) [78]. However, there is a lack of official documentation and academic papers dedicated explicitly to COI regarding the vaccine in infectious disease outbreaks. Our research primarily yielded news articles and expert critiques on this topic. During the COVID-19 pandemic in Iran, cases of COI emerged in various areas, including allocating funds to domestic vaccine producers, vaccine importation, and vaccine-related research. These COIs raised concerns about potential biases, fairness, and transparency in decision-making processes. However, despite these COIs, Iran has not implemented a comprehensive policy strategy to address them effectively. This lack of a robust policy approach to managing COIs may have impeded efforts to ensure fair and unbiased decision-making throughout the pandemic response [54, 62, 79].

Iranian Policymakers need to acknowledge the importance of managing COI regarding the vaccine in infectious disease outbreaks and prioritise developing and implementing appropriate policies and strategies.

Finally, it is essential to acknowledge the limitations of this study. Firstly, a scoping review methodology in this policy brief may narrow the available data coverage, limiting the overall understanding breadth. Additionally, it should be noted that while the optional consultation with stakeholders and experts for additional insights is recommended in the O'Malley and Arksey protocol, we have chosen not to pursue this step in our project. Consequently, there is a possibility of failing to fully capture the diverse perspectives of stakeholders involved in the decision-making process.

Lastly, including articles with average quality due to the limited availability of literature on the topic can introduce uncertainty or inconsistency in the findings and recommendations presented in the policy brief.

## Conclusion

Regarding the necessity of establishing relationships with the industry, it is crucial to take appropriate measures to address COI while fully recognising the COI that may arise from such relationships. In this regard, following sessions conducted by the research team and expert consultations and with consideration given to the principles underpinning good governance for health, the proposed policy options in this policy brief are summarised and prioritised as follows:

1. **Promoting disclosure and transparency of COI, standardisation of disclosure procedures, and subsequently, limitations on the involvement of members with COI** are directly aligned with the key principles of good governance for health, including “Transparency”, “The Rule of Law”, “Effectiveness and Efficiency”, “Responsibility”, and “Accountability”.
2. **Raising awareness among policymakers and researchers, fostering dialogue, and promoting shared discourse on COI** are directly aligned with the key principles of good governance for health, including “Consensus Orientation” “Participation”, “Effectiveness and Efficiency”.
3. **Enhancing the monitoring, evaluation, and accountability of policymakers and establishing appropriate penalties for any violations of the law** are directly aligned with the key components of good governance for health, including “Participation”, “Equality”, “The Rule of Law”, “Responsibility”, “Accountability”, “Transparency” and “Effectiveness and Efficiency”.
  It is essential to recognise that the findings and recommendations outlined in this policy brief may have context-specific implications and may not be directly applicable to different settings or regions. Therefore, we recommend conducting policy dialogues with stakeholders to discuss the policy options’ advantages, disadvantages, and practical considerations. Moreover, evaluating the effectiveness of these policy options in real-world scenarios is essential, as this evaluation may reveal the need for potential revisions to ensure practicality and efficacy.

### Supplementary Information

**Additional file 1: Appendix 1.** Search strategy.

**Additional file 2: Appendix 2.** PRISMA flow diagram.

## Data Availability

The datasets generated and/or analysed during the current study are not publicly available but are available from the corresponding author upon reasonable request.

## Abbreviations

COI: Conflict of interest
SID: Scientific Information Database
EMA: European Medicines Agency
OECD: Organisation for Economic Co-operation and Development

## Footnotes

1 The protocol consists of six stages: identification of research questions, identification of relevant articles through searches in reputable scientific databases, review of grey literature, theses, and relevant review articles, selection of relevant articles from retrieved primary studies, extraction of data into tables and charts, compilation, summarization, and dissemination of findings, and optional consultation with stakeholders and experts for additional insights.
