# Supplementary material for: Addressing conflicts of interest regarding the vaccine in infectious disease outbreaks based on good governance for health approach: a policy brief
Source: BMC Health Serv Res. 2023 Sep 25;23:1028. doi: 10.1186/s12913-023-10020-w (PMC10521481; doi:10.1186/s12913-023-10020-w)
Supplement: Supplementary file 1 — Additional file 1: Appendix 1. Search strategy. [file 12913_2023_10020_MOESM1_ESM.docx]

Appendix 1: Search Strategy

| PubMed | Pandemic OR Pandemics OR Epidemic OR Epidemics OR “Disease Outbreaks” OR “Disease Outbreak” OR (Outbreak AND Disease) OR (Outbreaks AND Disease) OR Outbreaks OR “Infectious Disease Outbreaks” OR (“Disease Outbreak” AND Infectious) OR (“Disease Outbreaks” AND Infectious) OR “Infectious Disease Outbreak” OR (Outbreak AND “Infectious Disease”) OR (Outbreaks AND “Infectious Disease”)  AND  “Conflict of Interest”[tiab] OR “Interest Conflict”[tiab] OR “Interest Conflicts”[tiab] OR Fraud OR Kickbacks OR Kickback OR “Drug Counterfeiting” OR (Counterfeiting AND Drug) OR “Health Care Fraud” OR “Healthcare Fraud” OR (Fraud AND Healthcare) OR (Fraud AND “Health Care”) OR “Health Care Frauds” OR Corruption  AND  Vaccine OR Vaccines |
| --- | --- |
| Scopus | ALL(Pandemic) OR ALL(Pandemics) OR ALL(Epidemic) OR ALL(Epidemics) OR ALL(“Disease Outbreaks”) OR ALL(“Disease Outbreak”) OR (ALL(Outbreak) AND ALL(Disease)) OR (ALL(Outbreaks) AND ALL(Disease)) OR ALL(Outbreaks) OR ALL(“Infectious Disease Outbreaks”) OR (ALL(“Disease Outbreak”) AND ALL(Infectious)) OR (ALL(“Disease Outbreaks”) AND ALL(Infectious)) OR ALL(“Infectious Disease Outbreak”) OR (ALL(Outbreak) AND ALL(“Infectious Disease”)) OR (ALL(Outbreaks) AND ALL(“Infectious Disease”))  AND  TITLE-ABS(“Conflict of Interest”) OR TITLE-ABS(“Interest Conflict”) OR TITLE-ABS(“Interest Conflicts”) OR ALL(Fraud) OR ALL(Kickbacks) OR ALL(Kickback) OR ALL(“Drug Counterfeiting”) OR (ALL(Counterfeiting) AND ALL(Drug)) OR ALL(“Health Care Fraud”) OR ALL(“Healthcare Fraud”) OR (ALL(Fraud) AND ALL(Healthcare)) OR (ALL(Fraud) AND ALL(“Health Care”)) OR ALL(“Health Care Frauds”) OR ALL(Corruption)  AND  ALL(Vaccine) OR ALL(Vaccines) |
| Embase | Pandemic OR Pandemics OR Epidemic OR Epidemics OR ‘Disease Outbreaks’ OR ‘Disease Outbreak’ OR (Outbreak AND Disease) OR (Outbreaks AND Disease) OR Outbreaks OR ‘Infectious Disease Outbreaks’ OR (‘Disease Outbreak’ AND Infectious) OR (‘Disease Outbreaks’ AND Infectious) OR ‘Infectious Disease Outbreak’ OR (Outbreak AND ‘Infectious Disease’) OR (Outbreaks AND ‘Infectious Disease’)  AND  ‘Conflict of Interest’:ti,ab OR ‘Interest Conflict’:ti,ab OR ‘Interest Conflicts’:ti,ab OR Fraud OR Kickbacks OR Kickback OR ‘Drug Counterfeiting’ OR (Counterfeiting AND Drug) OR ’Health Care Fraud’ OR ’Healthcare Fraud’ OR (Fraud AND Healthcare) OR (Fraud AND ’Health Care’) OR ’Health Care Frauds’ OR Corruption  AND  Vaccine OR Vaccines |
| WOS | ALL=(Pandemic) OR ALL=(Pandemics) OR ALL=(Epidemic) OR ALL=(Epidemics) OR ALL=(“Disease Outbreaks”) OR ALL=(“Disease Outbreak”) OR (ALL=(Outbreak) AND ALL=(Disease)) OR (ALL=(Outbreaks) AND ALL=(Disease)) OR ALL=(Outbreaks) OR ALL=(“Infectious Disease Outbreaks”) OR (ALL=(“Disease Outbreak”) AND ALL=(Infectious)) OR (ALL=(“Disease Outbreaks”) AND ALL=(Infectious)) OR ALL=(“Infectious Disease Outbreak”) OR (ALL=(Outbreak) AND ALL=(“Infectious Disease”)) OR (ALL=(Outbreaks) AND ALL=(“Infectious Disease”))  AND  TS=(“Conflict of Interest”) OR TS=(“Interest Conflict”) OR TS=(“Interest Conflicts”) OR ALL=(Fraud) OR ALL=(Kickbacks) OR ALL=(Kickback) OR ALL=(“Drug Counterfeiting”) OR (ALL=(Counterfeiting) AND ALL=(Drug)) OR ALL=(“Health Care Fraud”) OR ALL=(“Healthcare Fraud”) OR (ALL=(Fraud) AND ALL=(Healthcare)) OR (ALL=(Fraud) AND ALL=(“Health Care”)) OR ALL=(“Health Care Frauds”) OR ALL=(Corruption)  AND  ALL=(Vaccine) OR ALL=(Vaccines) |
