# Supplementary material for: Addressing conflicts of interest regarding the vaccine in infectious disease outbreaks based on good governance for health approach: a policy brief
Source: BMC Health Serv Res. 2023 Sep 25;23:1028. doi: 10.1186/s12913-023-10020-w (PMC10521481; doi:10.1186/s12913-023-10020-w)
Supplement: Supplementary file 2 — Additional file 2: Appendix 2. PRISMA flow diagram. [file 12913_2023_10020_MOESM2_ESM.doc]

Appendix 2: PRISMA flow diagram

**Screening**

**Included**

**Eligibility**

**Identification**

Records identified through database searching
(n = 1625)

Additional records identified through other sources
(n = 10)

Records after duplicates removed
(n = 1555)

Records screened
(n = 1555)

Records excluded
(n = 1332)

Full-text articles assessed for eligibility
(n = 223 )

Full-text articles excluded, with reasons
(n = 180 )

Studies included
(n =43)
